# Electric Field-Modulated Surface Enhanced Raman Spectroscopy by PVDF/Ag Hybrid

**DOI:** 10.1038/s41598-020-62251-0

**Published:** 2020-03-24

**Authors:** Jiajun Lu, Yuzhi Song, Fengcai Lei, Xuejian Du, Yanyan Huo, Shicai Xu, Chonghui Li, Tingyin Ning, Jing Yu, Chao Zhang

**Affiliations:** 1 Collaborative Innovation Center of Light Manipulations and Applications & Institute of Materials and Clean Energy, School of Physics and Electronics, Shandong Normal University, Jinan, 250014 P.R. China; 2 College of Chemistry, Chemical Engineering and Materials Science, Shandong Normal University, Jinan, 250014 P.R. China; 3 Shandong Key Laboratory of Biophysics, College of Physics and Electronic Information, Institute of Biophysics, Dezhou University, Dezhou, 253023 China

**Keywords:** Optical physics, Nanophotonics and plasmonics

## Abstract

Electrically modulated surface enhanced Raman scattering (E-SERS) can be able to regulate the plasmon resonance peak of metal nanostructures, further improve the detection sensitivity of the SERS substrate. However, the E-SERS substrates require auxiliary equipment to provide the electrical potential, and most of them are non-flexible structure, which limits the application of E-SERS in the portable, *in-situ* and fast detection area. Here, we developed an electric field-modulated SERS substrate based on the piezoelectric effect by combining the PVDF (piezoelectric-modulated layer) and Ag nanowires (AgNWs) (SERS active layer) and investigated the SERS activity in experiment and theory. The enhanced electric field and the tunable plasmon resonance induced by the piezoelectric effect provide the additional enhancement for the SERS signal. Furthermore, we fabricated a SERS active ring with a piezoelectric field-modulated substrate and achieved the *in-situ* detection of glucose with a non-invasive method. This work provided innovation for the E-SERS and could greatly promote the development of the *in-situ*, wearable and intelligent sensors.

## Introduction

Surface-enhanced Raman spectroscopy (SERS)^1–3^ which combines molecular fingerprint specificity and potential single-molecule sensitivity, has been widely used in surface science, electrochemistry, biology, materials science and other fields due to its high sensitivity and extremely fast response^4–8^. It is known that the SERS enhancement effect is caused by electromagnetic mechanism (EM)^9–12^ and chemical mechanism (CM)^13–15^, which are ascribed to the enhancements of local electric fields with the assist of the surface plasmon resonance and charge transfer, respectively. In recent years, how to prepare SERS substrates with excellent activity by simple methods has captured increasing attention^16–19^. Among numerous SERS techniques, the electrically modulated SERS (E-SERS) technology is particularly noteworthy by the virtue of the versatile ability to regulate the plasmon resonance of metal nanostructures through the electric field^20–23^. Besides, by adjusting the electrical signal, the E-SERS can be utilized to distinguish and investigate the corresponding enhancement mechanisms (EM or CM) in complex spectra^24–26^. The key factor that limits the rapid development of E-SERS technology is the complex equipment, where the measurements are mostly carried out in the sample cell. In addition, the demand for the SERS substrate is relatively special. The substrate should not only possess the nano-scale topography with enhanced activity but also with the excellent conductive properties, greatly hindering the rapid and *in-situ* detection^27^.

The piezoelectric effect discovered in 1880^28^, can produce the opposite charges on the surface with the interaction of mechanical force and has aroused much attention. Among the numerous piezoelectric materials, flexible polyvinylidene fluoride (PVDF) with remarkable piezoelectric effect has been widely researched as the representative material^29,30^. Compared with the scheme to conduct the flexible insulative polymer, the PVDF can provide an internal electric field under the pressure, which can serve as an ideal E-SERS substrate^31,32^. Besides the excellent flexibility for *in-situ* detection, the low Raman cross-section of the PVDF makes it has little influence on the identification of the probe molecular. Whereas, up to now, most of the reported researches on the PVDF SERS focus on the flexibility but ignore its piezoelectric effect^32^. Thus, to provide a deep and detail insight into the hidden light in force in the E-SERS, especially for the piezoelectric effect modulated Raman spectroscopy, is crucial and beneficial to better understand the enhancement mechanism.

**Figure 1:**
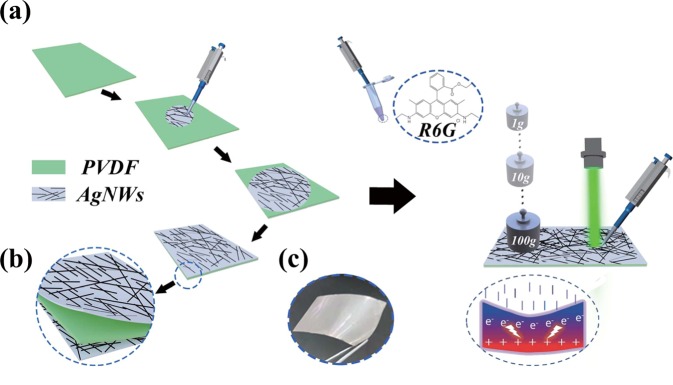
(**a**) The preparation and SERS measurement processes of the flexible piezoelectric substrate. (**b**) Schematic illustration of the three-layer SERS structure. (**c**) Photo of the flexible piezoelectric substrate.

Inspired by this, in this paper, we developed an electric field-modulated Raman substrate based on the piezoelectric effect by combining the PVDF (piezoelectric-modulated layer) and Ag nanowires (AgNWs) (SERS active layer). As exhibited in Fig. 1(a), the crisscrossed AgNWs are deposited on both sides of PVDF film with a simple spin coating method, in order to facilitate the integration with other devices, such as wearable device^33^. The top-layer crisscrossed AgNWs here act as the SERS active layer and can excite and produce the hot spots from the in-plane and interfacial AgNWs with the assist of the light-induced plasmonic resonances. The bottom-layer crisscrossed AgNWs are not absolutely required for the electric field-modulated SERS substrate but can serve as a flexible conductive electrode combining with the top-layer one. The pivotal point of the designed electric field-modulated SERS substrate [Fig. 1(b)] is the introduction of the flexible piezoelectric-modulated PVDF layer, where the internal electric field produced under the pressure can effectively regulate the surface plasmonic property of the AgNWs and further modulate the distribution of the hot spots around the AgNWs. This work can provide innovation for the E-SERS and will greatly promote the development of the *in-situ*, wearable and intelligent sensors.

## Materials and Methods

### Preparation of AgNWs/PVDF/AgNWs

The silver nanowire (synthesized by a solvothermal synthesis method) solution (diameter: 60 nm, length: 20 μm) was dispersed in ethyl alcohol was ultrasonically treated for 20 minutes. Then the PVDF film with a thickness of 10 μm was cleaned with ethyl alcohol to remove dust and impurities. After that, the AgNWs were evenly coated on both sides of the PVDF film with a Spin coating method. To test the piezoelectric effect of the substrate, the conductive copper tape was bonded on all sides of the AgNWs/PVDF/AgNWs and the acoustic response was recorded by an audio analyzer (U8903A).

### Raman detections

Raman spectra were collected with a Horiba HR Evolution 800 Raman microscope system with a laser of 532 nm under the power of 0.048 mW, and the diffraction grid and integration time were respectively set as 600 gr/mm and 8 s throughout the experiment.

### FDTD Simulations

In the FDTD simulations, the Drude-Lorentz (DL) model was considered in the simulation. According to the DL model, metal’s permittivity can be written as

1 $$\varepsilon (\omega )={\varepsilon }_{\infty }-\frac{{\omega }_{p}^{2}}{{\omega }^{2}+i{\gamma }_{D}\omega }-\frac{f{{\Omega }}_{L}^{2}}{{\omega }^{2}-{{\Omega }}_{L}^{2}+i\omega {\gamma }_{L}}$$

where *ε*_∞_ is the frequency-independent dielectric constant, *ω* and *ω*_*p*_ are respectively the frequency of incident light and the plasma frequency of the metal, Ω_*L*_ represents the oscillator strength of Lorentz oscillators, which is proportional to the *ω*_*p*_ (Ω_*L*_ = *c*_0_*ω*_*p*_, where *c*_0_ is a constant). *f* can be interpreted as the Lorentz weighting, and *γ*_*D*_ *γ*_*L*_ are the respectively the damping constants related to free and bound electrons. One interesting thing is that in this model the *ε*(*ω*) has a close relationship with the electron density *N*, due to that the *ω*_*p*_ can be represented as:

2 $${\omega }_{p}^{2}=\frac{N{e}^{2}}{{\varepsilon }_{0}{m}_{e}}$$

where *e*, *ε*_0_ and *m*_*e*_ are electron charge, vacuum permittivity and effective mass of electrons, respectively.

In our experiments, partial Ag NWs nearby the PVDF can discharge due to the transfer of an electron under the influence of positive potential by piezoelectric effect^31,34,35^. The decreased electrons in Ag NWs subsequently lead to the change of the *ε*(*ω*) owing to the alteration of *ω*_*p*_ and Ω_*L*_, represented as

3 $${\omega }_{p}^{\text{'}}={\omega }_{p}\sqrt{1+\frac{\varDelta N}{N}}$$

4 $${\varOmega }_{L}^{{\prime} }={\varOmega }_{L}\sqrt{1+\frac{\varDelta N}{N}}$$

where ${\omega }_{p}^{{\prime} }$ and ${\varOmega }_{L}^{{\prime} }$ are the changed plasma frequency and oscillator strength of Lorentz oscillators, and Δ*N* is the changed electron density. In our simulation, the values of all the parameters that appeared in Eqs. (1) to (4) were obtained from refs. ^31,34–36^.

In the simulation, two orthogonal Ag NWs with a length of 1 µm and a diameter of 60 nm were adopted, and the gap between them was set as 5 nm according to the SEM image. Incident light and monitoring light were both set as 532 nm in the simulation of electric field distribution. And to achieve the simulated LSPR peak of Ag NWs by absorption cross-section, incident lights from 300 nm to 700 nm were used.

## Results and Discussion

**Figure 2:**
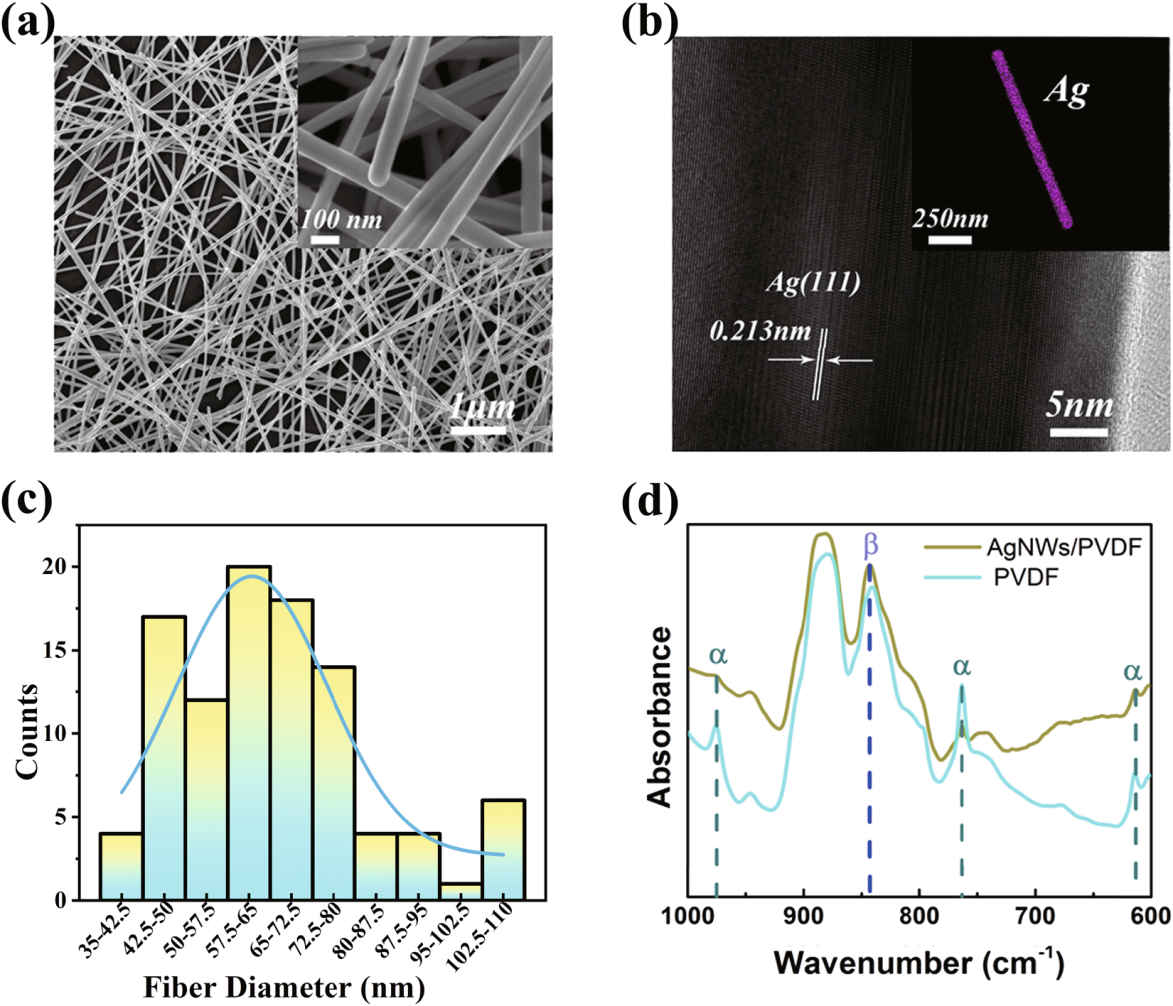
(**a**) SEM image of PVDF covered by AgNWs. (Inset: SEM image of PVDF covered by AgNWs at larger magnification.) (**b**) The HRTEM image of AgNWs (Inset: EDS image of Ag element.) (**c**) Distribution of the diameter for AgNWs. (**d**) FTIR spectrum of pure PVDF and PVDF covered by AgNWs.

The optical and scanning electron microscope (SEM) image of the pure PVDF film was shown in Fig. S1, and the AgNWs SEM image in Fig. 2(a) clearly exhibits the crisscrossed structure of the single nanowire with the length up to 20 μm and the diameter ca. 60 nm. What should be noted here is that the crisscrossed structure is not only the in-plane intersection but also the interfacial intersection forming a multidimensional coupling system. The clear and well-distributed Ag element appears in the energy dispersive spectrometry (EDS) mapping in the inset of Fig. 2(b) demonstrating the successful fabrication of the AgNWs. The high-resolution transmission electron microscope (HRTEM) of the AgNWs in Fig. 2(b) exhibits 0.231 nm lattice fringe spacing corresponding to the (111) planes, combined with the distinct and bright six fold symmetric symmetry pot pattern in selected area electron diffraction (SAED) [see Fig. S2], proving the high crystallinity of the AgNWs. The distribution of the diameter for AgNWs is shown in Fig. 2(c). As shown in Fig. S3, the absorption spectrum indicates that the LSPR peaks of the pristine AgNWs with the main peak at 378 nm and a shoulder at 350 nm. The Fourier transform infrared spectrometer (FTIR) spectrum of the PVDF and AgNWs/PVDF were collected as shown in Fig. 2(d). The α and β phase are co-existed and the latter one is responsible for the piezoelectric response of the PVDF film. The content of phase β PVDF increases after the addition of AgNWs, combined with the good acoustic response collected experimentally [Fig. S4], demonstrating the promotion of piezoelectric response.

**Figure 3:**
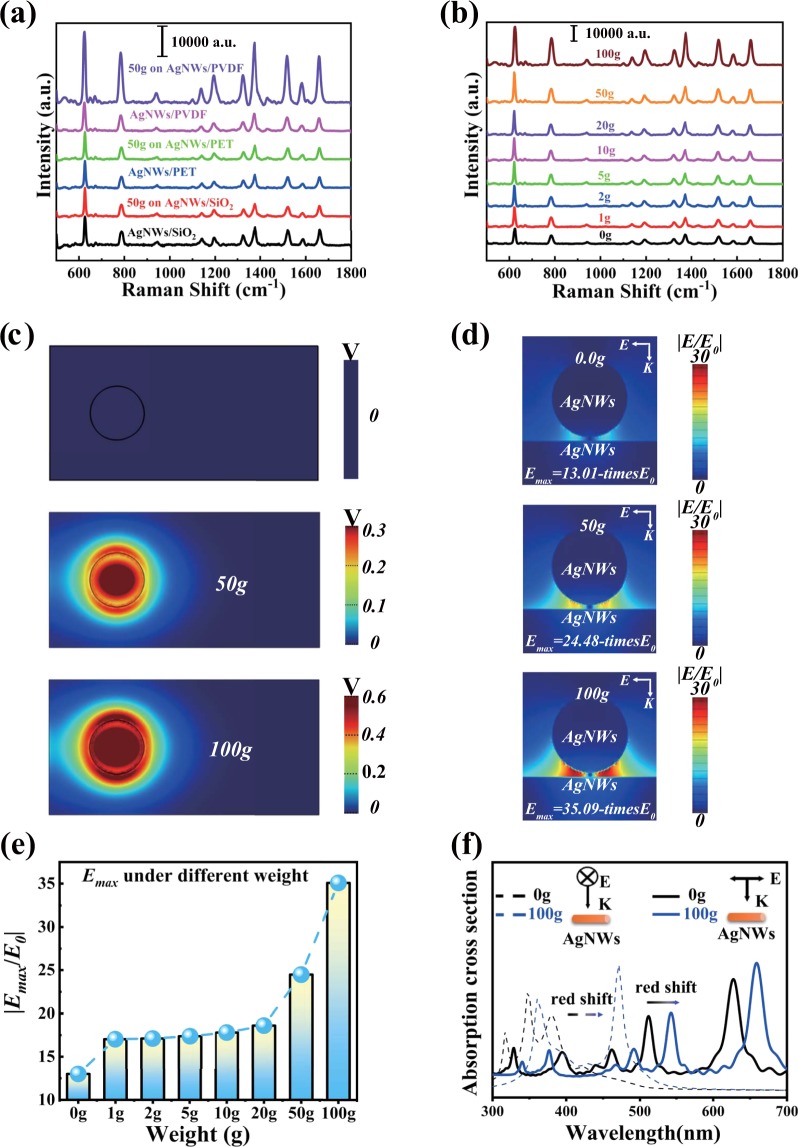
(**a**) The SERS signals of the R6G with the concentration of 10^−6^ M on the different substrates with or without of the 50 g weight. (**b**) SERS signals of R6G with a concentration of 10^−6^ M under different weights. (**c**) Potential distribution of the PVDF under different pressure. (**d**) Electric field distributions of the AgNWs under different potential. (**e**) Correlation between the maximum electric field and weight. (**f**) Absorption cross-section of the AgNWs without and with the 100 g weight.

The R6G molecular with the concentration of 10^–6^ M was chosen to investigate the electric field-modulated SERS activity of the fabricated AgNWs/PVDF substrate in this work. We can see clearly in Fig. 3(a) that the intensity of the SERS signal for R6G on the AgNWs/PVDF substrate increase obviously when dropped a 50 g weight on its surface. By contrast, under the same condition, the enhancements for the SERS signal on the normal flexible AgNWs/PET and rigid AgNWs/SiO_2_ substrate are almost ignorable, which demonstrates that the increase of SERS signal on the AgNWs/PVDF substrate may be contributed to some changes introduced by the weight drop. [Fig. S5 presents the corresponding Raman spectra of the R6G on the pure PVDF, PET and SiO_2_ substrate].

In order to explore the effect of different pressures on the substrate, the enhancement of SERS signal correlation to different pressures on the substrates was further explored. We collected the SERS spectra of the R6G on the AgNWs/PVDF substrate under different weights (from 1 to 100 g), where all the weights were placed on the same position of the substrates. As exhibited in Fig. 3(b), with the increase of the weight, the intensity of the SERS signal increase obviously, which indicates the obvious pressure effects on the Raman activity. To gain a more convenient observation of the spectral changes, the comparisons of the peak at 613 cm^−1^ under different weights were shown in Fig. S6.

To give a deep insight into this phenomenon, the potential distribution of PVDF films under different pressures were simulated firstly using the FDTD method. From Fig. 3(c), we found that a positive potential around the weight appeared on the surface after placed a weight. The more pressures we put on the PVDF materials, the greater potential was generated. Next, the electric fields nearby the Ag NWs under different pressures were obtained as shown in Fig. 3(d). The electric field in the gap area among the AgNWs was greatly enhanced compared with the sample without weight using the 532 nm incident laser. We suspect that this phenomenon is caused by the charge transfer between the piezoelectric layer and the plasmonic structure. The AgNWs nearby the PVDF can be charged under an electrostatic induction of the positive potential^27,30^. As shown in Fig. 3(e), with the increase of the weight, the number of transferred electrons of the AgNWs keeps increasing, which would further enhance the electric field for EM. Thus, the maximum intensity of the electric field in the gap between two adjacent Ag NWs improved from 13.01 to 35.09. The more information about the potential distribution of PVDF films and the electric field strength of the SERS substrate can be seen in Figs. S7 and S8.

What’s more, as shown in Fig. 3(f), these induced electrons bring a change to ε(ω) and further lead to a redshift of the plasmon resonance of the AgNWs, which better matching with the incident laser (532 nm) used in the Raman measurement and benefits the regulation of the LSP property of the SERS substrate^31,35,36^.

According to the fourth power law, the EF of the SERS substrate with the wight of 100 g can reach 1.5 × 10^6^. Based on the discussions above, it is no less reasonable to believe that the SERS enhancement for the AgNWs/PVDF substrate can be attributed to the enhanced electric field and the tunable SPR induced by the piezoelectric effect.

In addition, we also found that the arrangement of AgNWs can be divided into two types: in-plane intersected and multidimensionally crisscrossed. The electric field analysis for these two cases is exhibited in Fig. S9. By contrast, only lateral hot spots exist in the former structure, however, the latter possess both the vertical and lateral hot spots for the multidimensionally crisscrossed structure. The SERS performance of the two structures was also tested experimentally. The SERS signal from the multidimensionally crisscrossed structure is much stronger than that from the in-plane intersecting AgNWs substrates as shown in Fig. S10.

**Figure 4:**
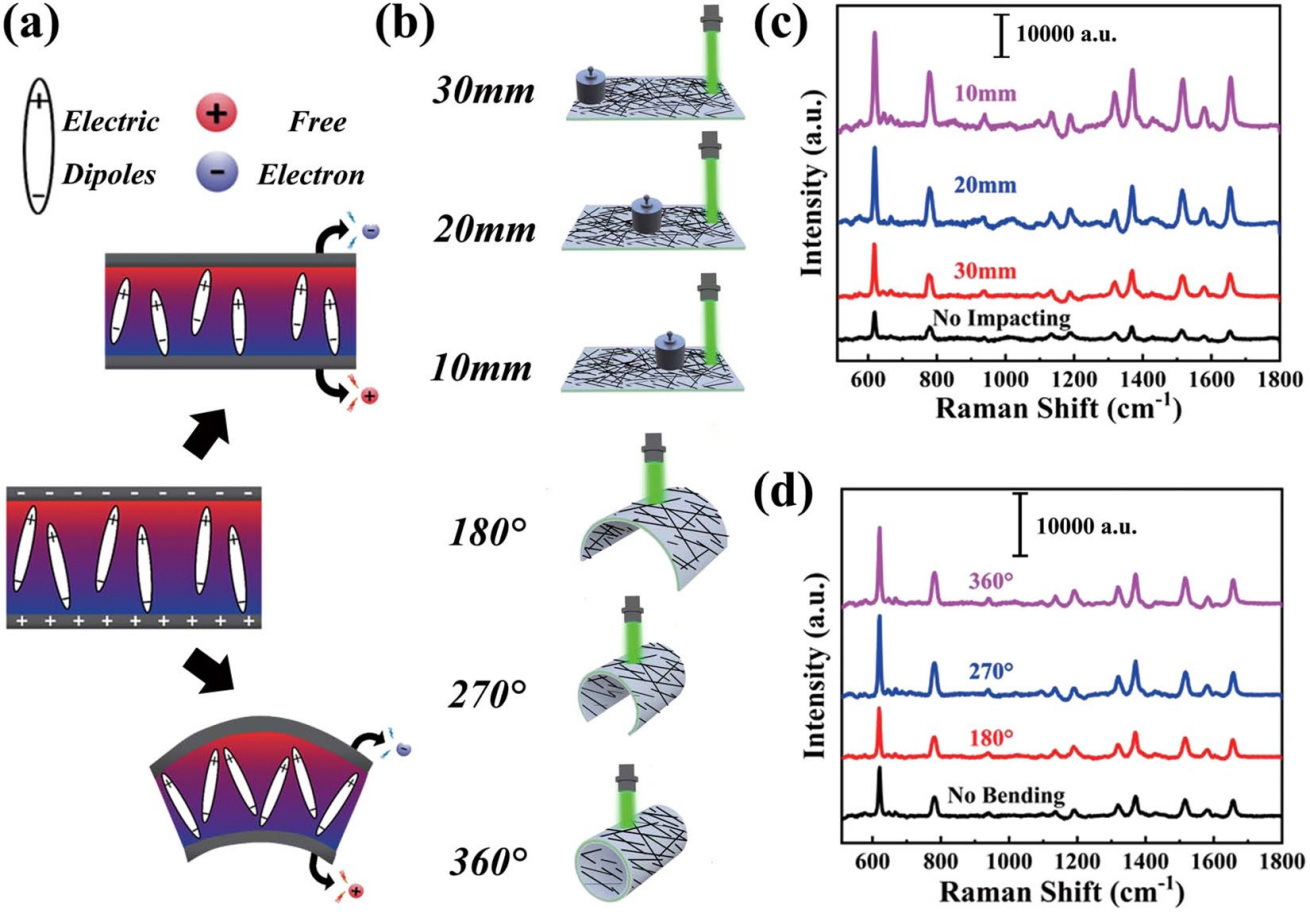
(**a**) The Piezoelectric principle diagram. (**b**) The schematic diagram of the SERS measurement. (**c**) The SERS signals of R6G with a concentration of 10^−6^ M under the weight of 50 g placed in different positions. (**d**) The SERS signals of the R6G with the same concentration under different bending.

Figure 4(a) exhibits the schematic illustration of the piezoelectric effect. The mechanical deformation introduced by impact or bend will create the piezoelectric potential (field). Here, we carried out a detailed investigation of the distance and bend dependence for the SERS signals. The process of the SERS measurement is schematically represented in Fig. 4(b). With the distance between the weight and the incident laser spot increase from 10 to 30 mm, the intensities of the peaks obviously decay [as shown in Fig. 4(c) and Fig. S11]. Further increasing the distance to 40 mm, the intensity further decreases and is similar to that no impacting. The reason account for this phenomenon is that the piezoelectric potential decays as the distance increases, shown in Fig. S12. The difference of the piezoelectric potential on different positions will lead to the diversity of the number of electrons of the AgNWs, which will further produce an effect on the electric field and the plasmon resonance of the AgNWs as discussed in Fig. 3. What’s more, interestingly, in Fig. 4(d) and Fig. S13, with the radius of curvature increase of the AgNWs/PVDF substrate, the SERS activity of the substrate is enhanced 2.5 times compared with that no bending, which can attributed to the piezoelectric potential introduced by the end of the substrate and is further discussed in the following section.

**Figure 5:**
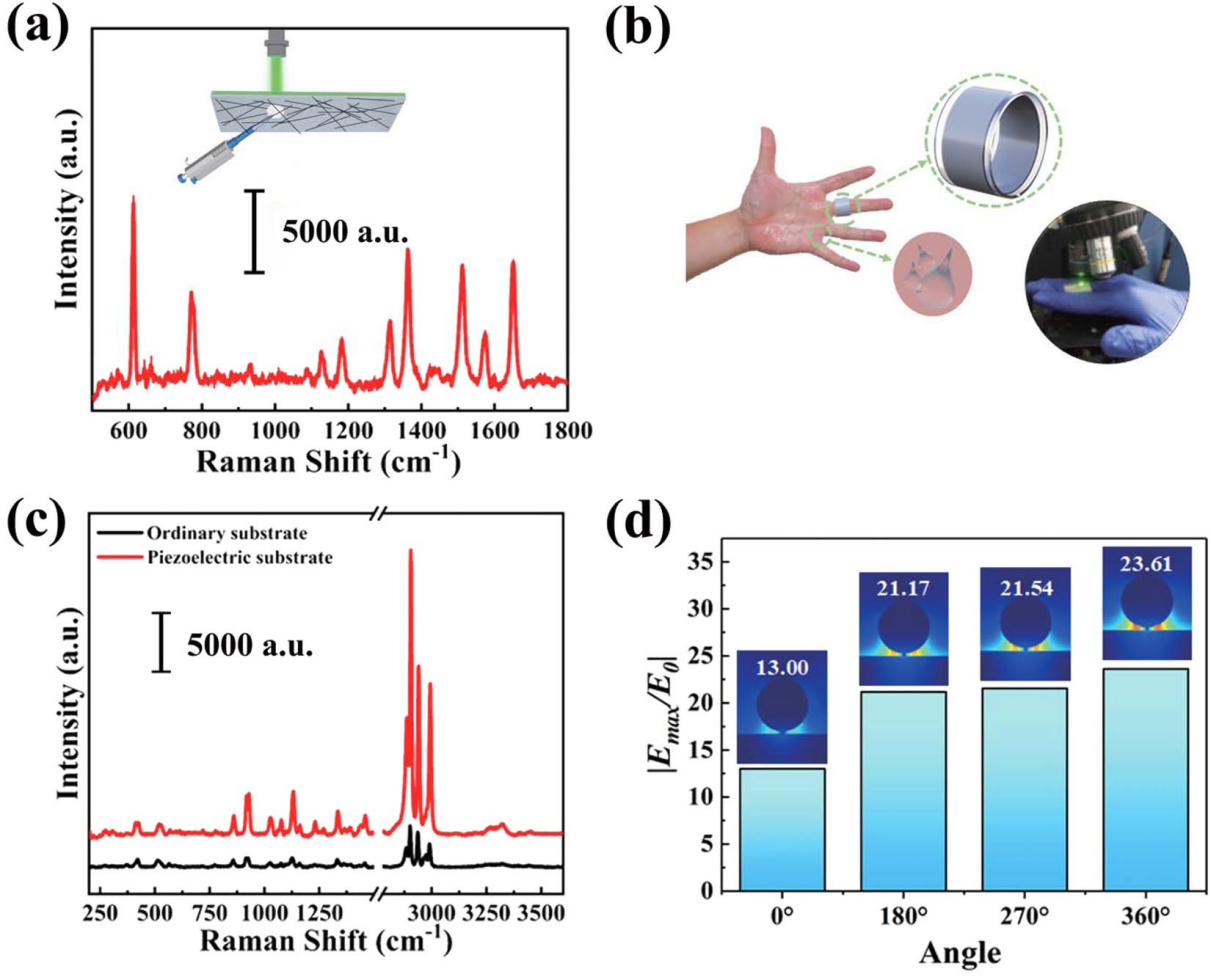
(**a**) The SERS signals of the R6G in the case of *in-situ* detection. (inset: schematic diagram of the *in-situ* SERS measurement). (**b**) The schematic diagram of the SERS ring. (**c**) The SERS signals of glucose collected with SERS ring and ordinary substrate. (**d**) The enhancement of the electric field under different bend.

By the virtue of the excellent flexibility and high transmissivity of the PVDF film, the designed piezoelectric electric field-modulated substrate has great potential for *in-situ* detection [inset in Fig. 4(a)]. The incident laser can pass through the PVDF, excite the hot spots around the AgNWs and further enhance the SERS signal of R6G in Fig. 5(a). Detection of glucose in sweat is the essential method to monitor the states of diabetes. Furthermore, as schematically shown in Fig. 5(b), we fabricated a SERS active ring with the AgNWs/PVDF substrate and achieved the *in-situ* detection of glucose with a concentration of 20% in Fig. 5(c) by a non-invasive method. Figure 5(d) presents the enhancement of the electric field distribution of the designed SERS ring under different bend, which indicates the promising prospects of the SERS ring for *in-situ* detection.

## Conclusions

In summary, a piezoelectric field-modulated Raman substrate with the piezoelectric effect by combining the PVDF and AgNWs is investigated experimentally and theoretically. The enhanced electric field and the tunable plasmon resonance induced by the piezoelectric effect are demonstrated as the reason for the additional SERS enhancement. This work presents promising prospects of the E-SERS and will greatly promote the development of the *in-situ*, wearable and intelligent sensors.

## Supporting Information

Supplementary Figures
