# Supplementary material for: Electric Field-Modulated Surface Enhanced Raman Spectroscopy by PVDF/Ag Hybrid
Source: Sci Rep. 2020 Mar 24;10:5269. doi: 10.1038/s41598-020-62251-0 (PMC7093541; doi:10.1038/s41598-020-62251-0)
Supplement: Supplementary file 1 — Supplementary Figures [file 41598_2020_62251_MOESM1_ESM.docx]

Electric Field-Modulated Surface Enhanced Raman Spectroscopy by PVDF/Ag Hybrid

Jiajun Lu ^1^, Yuzhi Song ^1,4^, Fengcai Lei ^2^, Xuejian Du ^1^, Yanyan Huo ^1^, Shicai Xu ^3^, Chonghui Li ^1^, Tingyin Ning ^1^, Jing Yu ^1,5^ and Chao Zhang ^1,^*

^1^ Collaborative Innovation Center of Light Manipulations and Applications & Institute of Materials and Clean Energy, School of Physics and Electronics, Shandong Normal University, Jinan 250014, P.R.China

^2^ College of Chemistry, Chemical Engineering and Materials Science, Shandong Normal University, Jinan 250014, P.R. China

^3^ Shandong Key Laboratory of Biophysics, College of Physics and Electronic Information, Institute of Biophysics, Dezhou University, Dezhou 253023, China

^4^ yzsong@sdnu.edu.cn

^5^ yujing1608@sdnu.edu.cn

***** [czsdnu@126.com](mailto:czsdnu@126.com)

**Supplementary information**


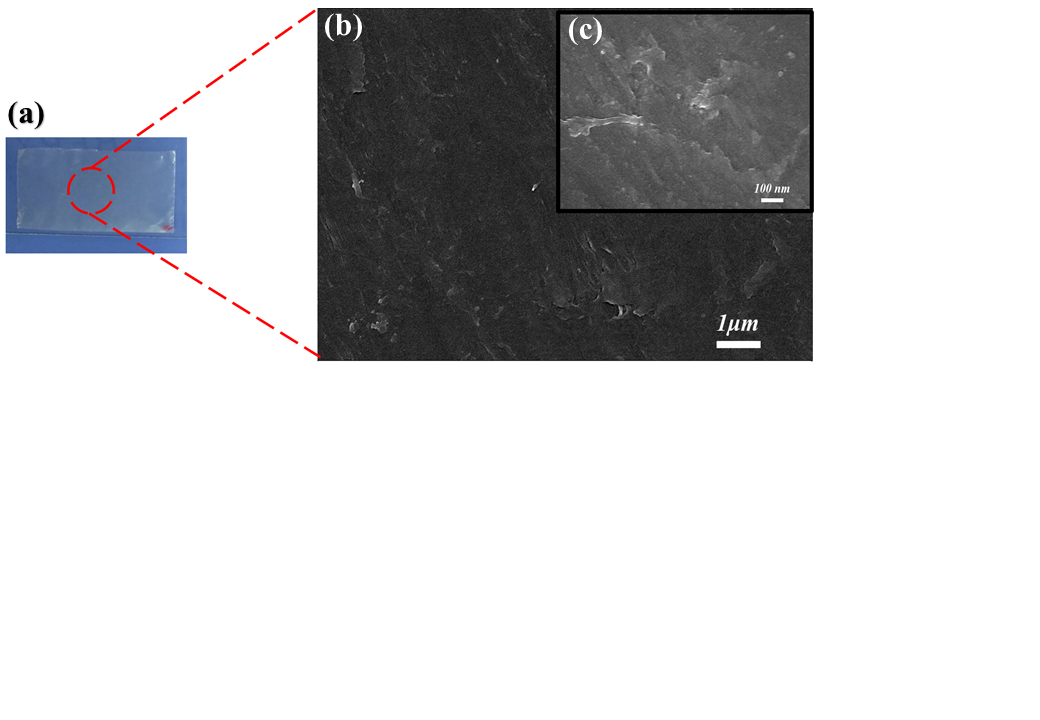


**Figure S1.** (a) Optical image of pure PVDF, (b) and (c) the SEM images of the pure PVDF under different magnifications.


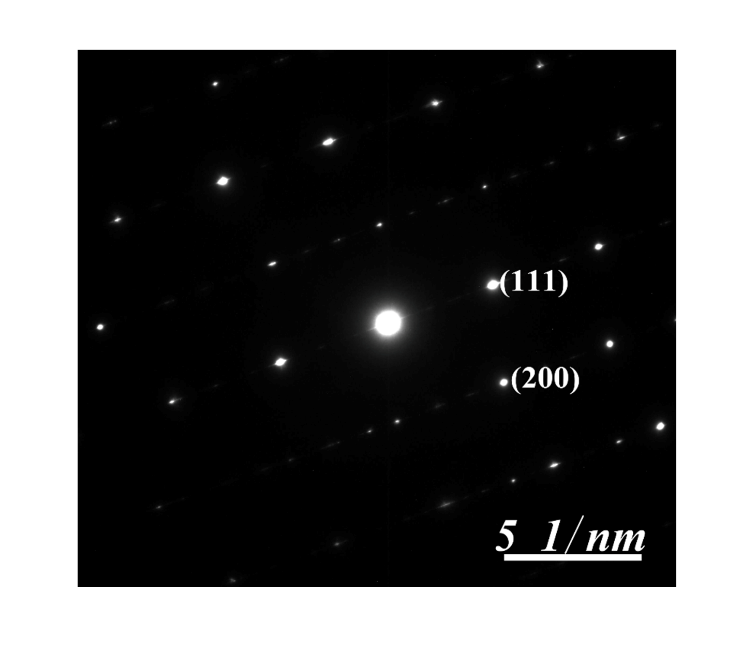


**Figure S2.** The SAED pattern of the AgNWs.





**Figure S3.** Absorption spectrum of the AgNWs.


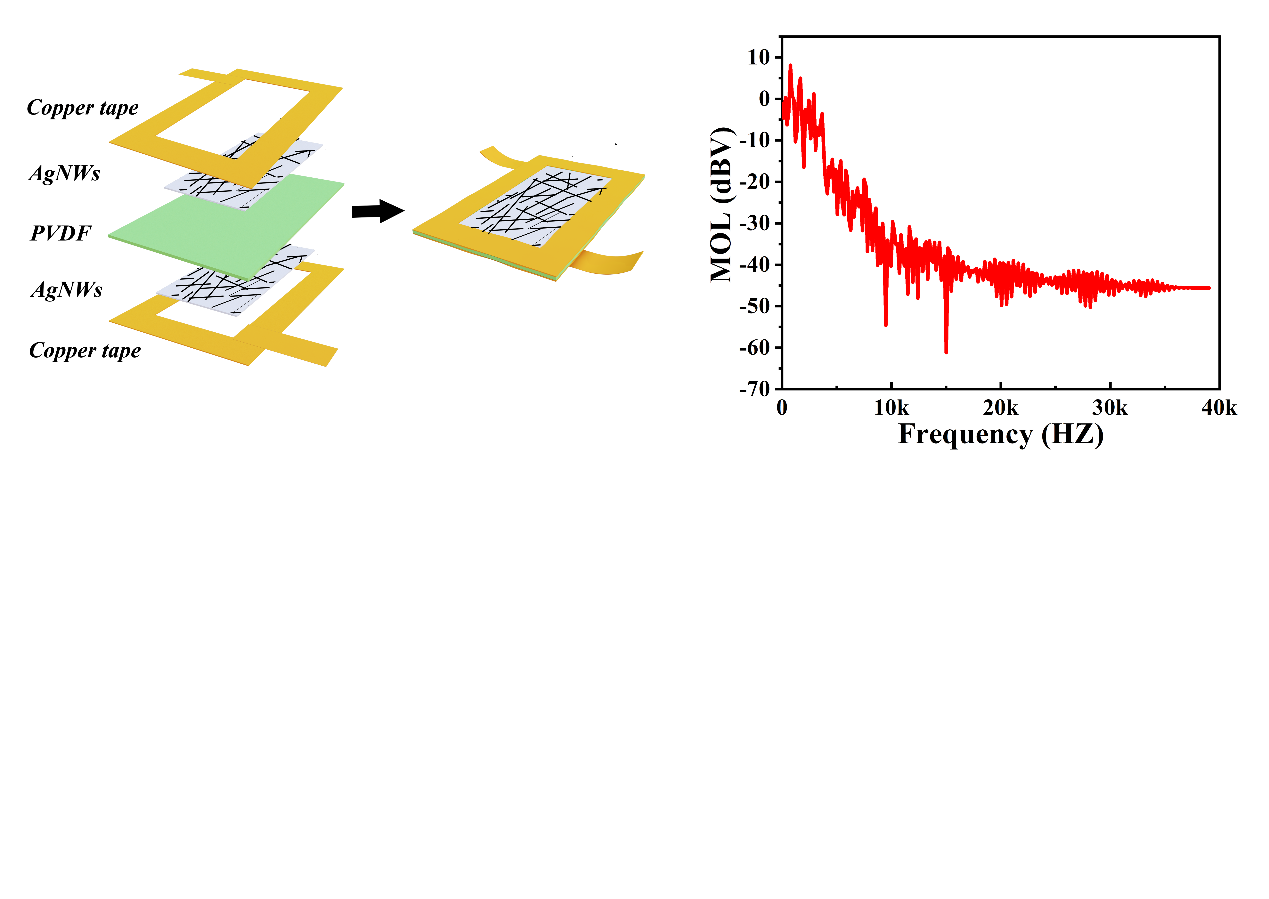


**Figure S4**. Audio response of the loudspeaker based on the AgNWs/PVDF/AgNWs





**Figure S5.** Raman spectra of R6G (10^-6^ M) detected by pure PVDF, SiO_2_, PET respectively.





**Figure S6.**Intensity comparisons of the peak at 613 cm^-1^ under different weights and corresponding enhancement.


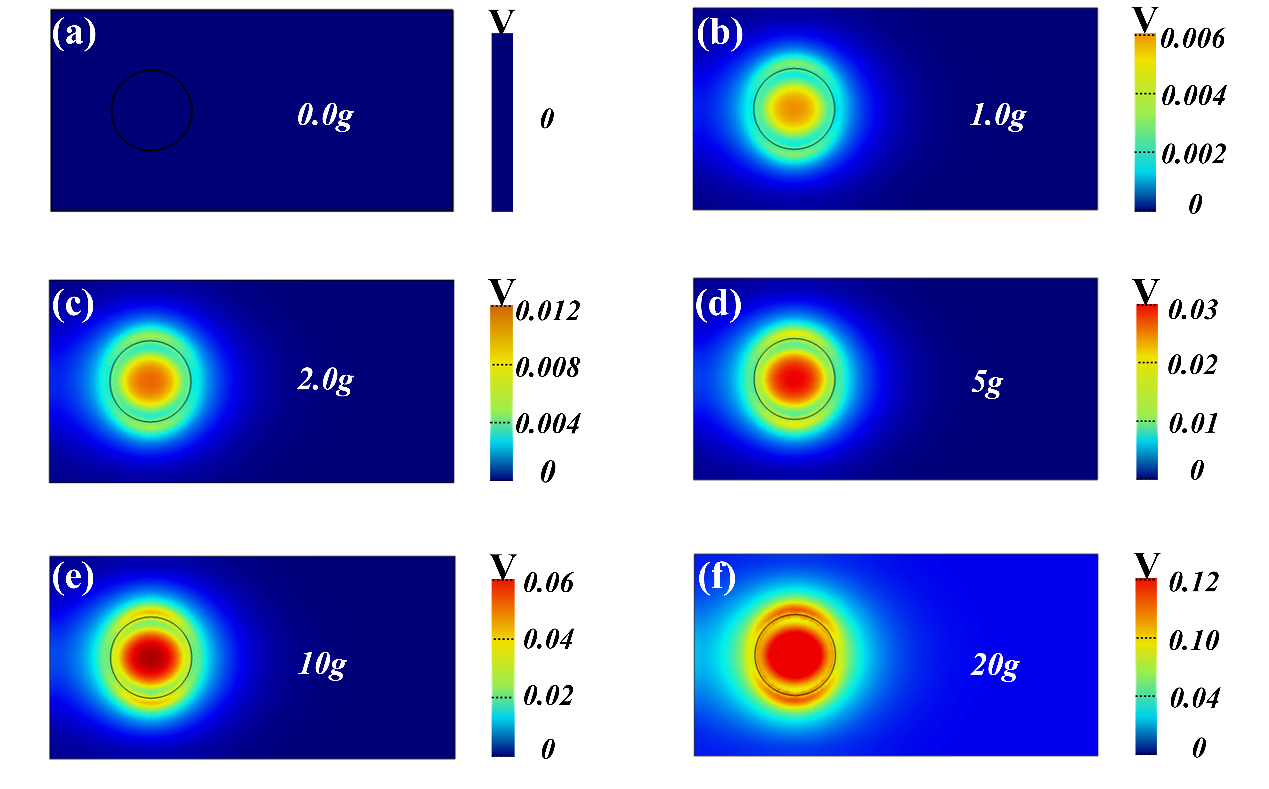


**Figure S7.** Potential distribution of the substrate under the pressure of different weights.


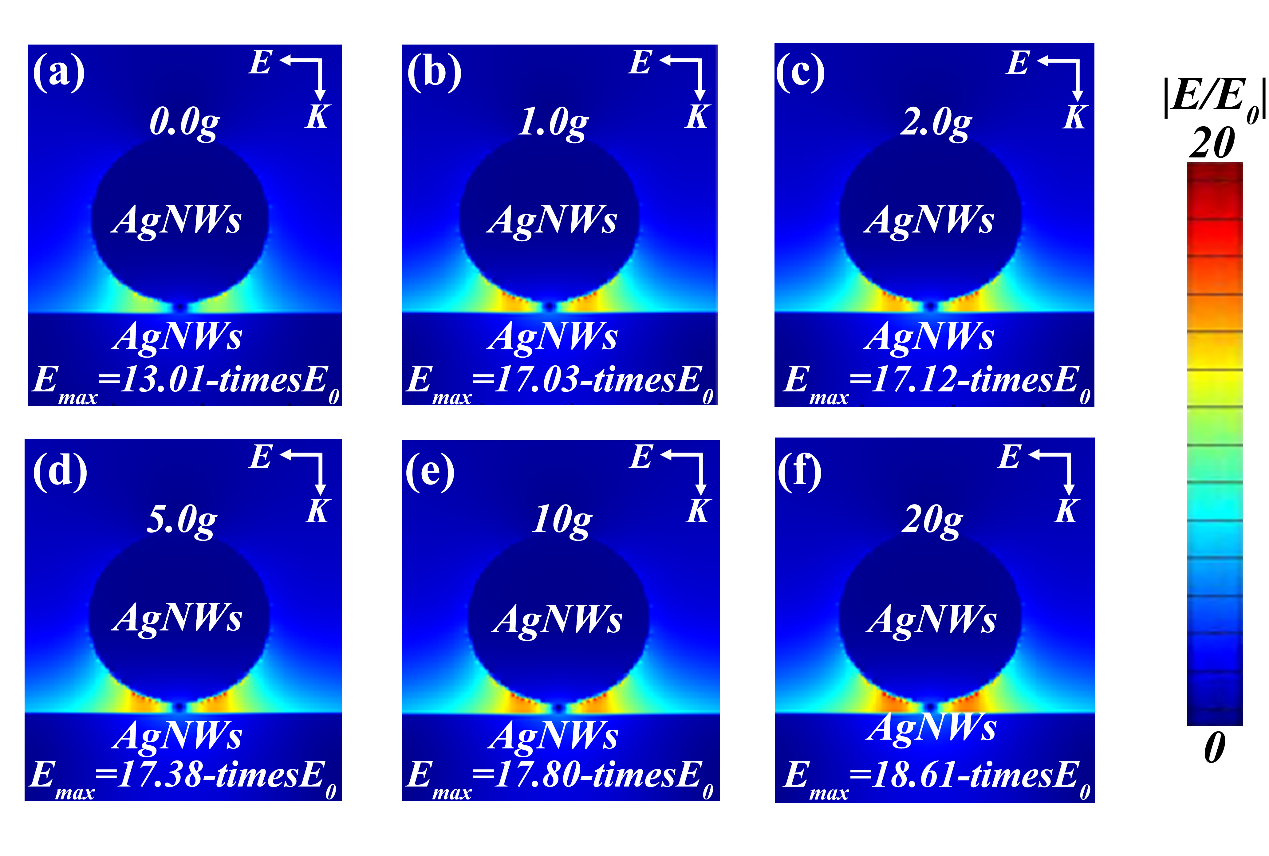


**Figure S8.** Electric field distributions of the AgNWs under different potential.


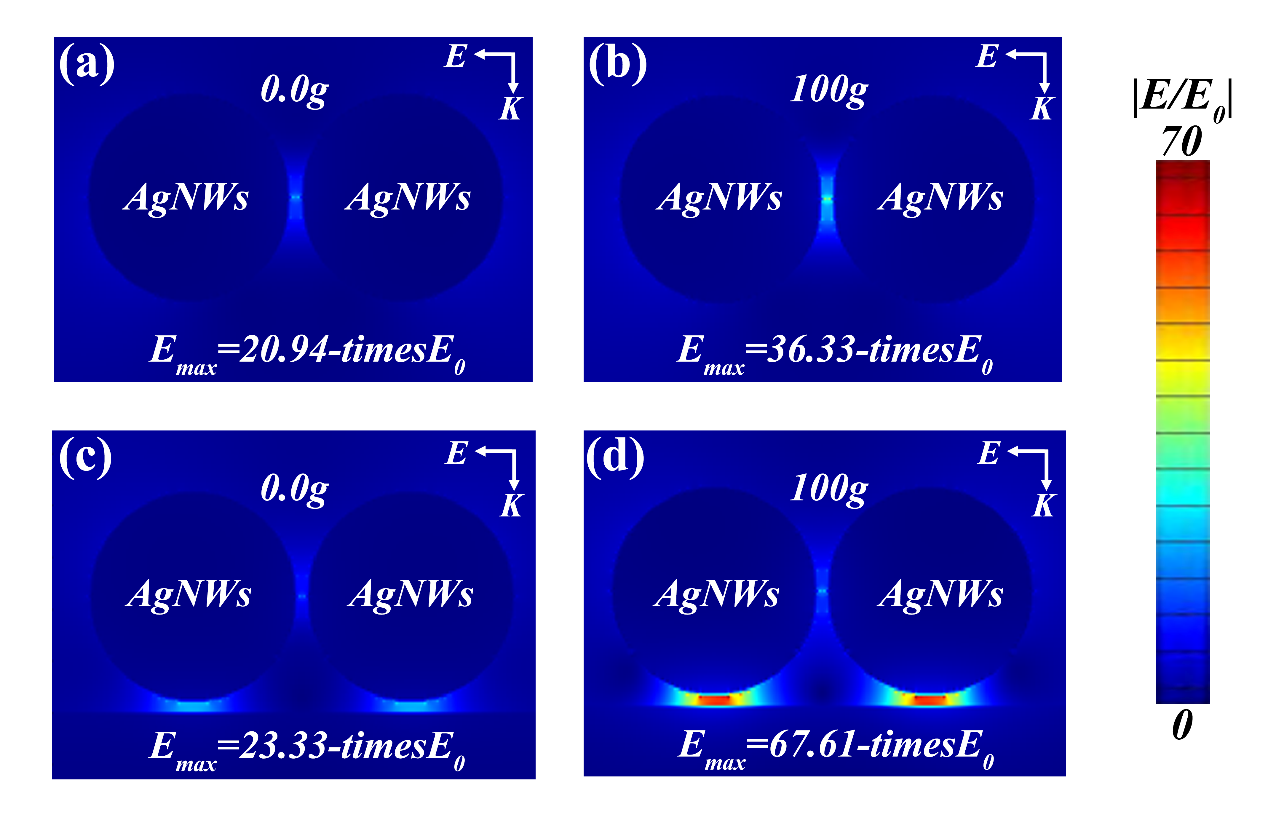


**Figure S9.** Electric field distributions of the (a) in-plane intersected and (b) multidimensionally crisscrossed AgNWs/PVDF substrate.





**Figure S10.** Raman spectra of R6G (10^-6^ M) detected on the multidimensionally crisscrossed and in-plane intersected AgNWs/PVDF substrate.

|   **Figure S11.** The peak intensities of 613cm^-1^ under the same weight in different positions. |
| --- |

|   **Figure S12.** The potential distribution of the substrate under the weight of 50g. |
| --- |

|   **Figure S13.** The peak intensities of 613cm^-1^ under different bending. |
| --- |
